# Risk of sexual transmitted infection following bipolar disorder: a nationwide population-based cohort study

**DOI:** 10.18632/oncotarget.24780

**Published:** 2018-04-03

**Authors:** Shyh-Chyang Lee, Chang-Kuo Hu, Jeng-Hsiu Hung, Albert C. Yang, Shih-Jen Tsai, Min-Wei Huang, Li-Yu Hu, Cheng-Che Shen

**Affiliations:** ^1^ Department of Psychiatry & Center for Geriatrics and Gerontology, Kaohsiung Veterans General Hospital, Kaohsiung, Taiwan; ^2^ Department of Orthopedics, Chiayi Branch, Taichung Veterans General Hospital, Chiayi, Taiwan; ^3^ Department of Information Management, Chia Nan University of Pharmacy and Science, Tainan, Taiwan; ^4^ Division of Neurosurgery, Department of Surgery, Chiayi Branch, Taichung Veterans General Hospital, Chiayi, Taiwan; ^5^ Department of Obstetrics and Gynecology, Taipei Tzu Chi Hospital, Buddhist Tzu Chi Medical Foundation, New Taipei City, Taiwan; ^6^ School of Medicine, Tzu Chi University, Hualien, Taiwan; ^7^ Department of Psychiatry, Taipei Veterans General Hospital, Taipei, Taiwan; ^8^ Center for Dynamical Biomarkers and Translational Medicine, National Central University, Chungli, Taiwan; ^9^ School of Medicine, National Yang-Ming University, Taipei, Taiwan; ^10^ Department of Psychiatry, Chiayi Branch, Taichung Veterans General Hospital, Chiayi, Taiwan

**Keywords:** bipolar disorder, sexually transmitted infection, epidemiology, public health, nationwide study

## Abstract

**Background:**

Bipolar disorder is a severe mental disorder associated with functional and cognitive impairment. Numerous studies have investigated associations between sexually transmitted infections (STIs) and psychiatric illnesses. However, the results of these studies are controversial.

**Objective:**

We explored the association between bipolar disorder and the subsequent development of STIs, including human immunodeficiency virus infection; primary, secondary, and latent syphilis; genital warts; gonorrhea; chlamydial infection; and trichomoniasis.

**Results:**

The bipolar cohort consisted of 1293 patients, and the comparison cohort consisted of 5172 matched control subjects without bipolar disorder. The incidence of subsequent STIs (hazard ratio (HR) = 2.23, 95% confidence interval (CI) 1.68–2.96) was higher among the patients with bipolar disorder than in the comparison cohort. Furthermore, female gender is a risk factor for acquisition of STIs (HR = 2.36, 95% CI 1.73–4.89) among patients with bipolar disorder. For individual STIs, the results indicated that the patients with bipolar disorder exhibited a markedly higher risk for subsequently contracting syphilis, genital warts, and trichomoniasis.

**Conclusions:**

Bipolar disorder might increase the risk of subsequent newly diagnosed STIs, including syphilis, genital warts, and trichomoniasis. Clinicians should pay particular attention to STIs in patients with bipolar disorder. Patients with bipolar disorder, especially those with a history of high-risk sexual behaviors, should be routinely screened for STIs.

**Methods:**

We identified patients who were diagnosed with bipolar disorder in the Taiwan National Health Insurance Research Database. A comparison cohort was constructed of patients without bipolar disorder who were matched with the bipolar cohort according to age and gender. The occurrence of subsequent new-onset STIs was evaluated in both cohorts.

## INTRODUCTION

Bipolar disorder is a severe mental disorder associated with functional and cognitive impairment [1–3] and increased risk of suicide [4]. The lifetime prevalence of bipolar disorder is 2.1% in the general population [5], and bipolar disorder is the sixth leading cause of disability worldwide [6]. Bipolar disorder is characterized by the presence of manic or hypomanic episodes, often alternating with depressive episodes [7]. The manic or hypomanic episodes of bipolar disorder are characterized by symptoms including elated mood, increased energy, grandiosity, decreased need for sleep, impulsivity, and hypersexuality [7], and these symptoms often lead to risky behaviors, including risky sexual behaviors.

Sexually transmitted infections (STIs) are infections that are commonly spread through sexual behaviors. In 2015, approximately 1.1 billion people had STIs other than human immunodeficiency virus (HIV) infection [3], and such STIs resulted in 108,000 deaths in 2015 [8]. In the United States, millions of cases of STIs occur each year, resulting in substantial medical costs. A study indicated that the total lifetime direct medical cost for STIs in 2008 in the United States was $15.6 billion and that the burden of STIs would be even greater in the absence of STI prevention and control efforts [9]. Therefore, it is essential to identify the risk factors of STIs and arrange appropriate screening protocols for people with risk factors.

Numerous studies have described associations between STIs and psychiatric illnesses [10–18]. However, most of these studies were focused on HIV infection only, and few studies have been designed to investigate the associations between psychiatric illness and other STIs, such as syphilis, genital warts, gonorrhea, chlamydial infection, and trichomoniasis [10, 17]. Also, the results of these studies are controversial. For example, a study in the United States revealed that HIV infection was present in 1.2% of the psychiatric outpatients, approximately four times the occurrence of HIV infection in the general adult population [11]. However, another study in the United States demonstrated that the odds of new HIV infection among beneficiaries with or without severe mental illness did not differ significantly [14]. Furthermore, most of these study results were based on cross-sectional study designs and lacked a longitudinal perspective [10–13, 15–18].

In response to the lack of longitudinal studies concerning the association between bipolar disorder and the subsequent risk of STIs and on the basis of the hypothesis that bipolar disorder, a severe psychiatric illness related to hypersexuality and risky behaviors, might be associated with a higher risk for developing subsequent STIs, we designed a nationwide population-based retrospective cohort study to investigate the possible link between these two illnesses.

## RESULTS

Our study sample comprised 1293 patients with bipolar disorder and 5172 control subjects without bipolar disorder. The demographic and clinical variables of the bipolar and control cohorts are compared in Table 1. The median age of the patients was 42.3 years (interquartile range, 30.9–56.3 y), and the median follow-up durations of the bipolar and control cohorts were 10.68 and 11.02 years, respectively. A higher percentage of patients with bipolar disorder were observed in the group of people aged 20–39 years. The baseline difference in comorbidities demonstrated a higher prevalence of hypertension, diabetes mellitus, dyslipidemia, coronary artery disease, congestive heart failure, chronic pulmonary disease, and cerebrovascular disease among the patients with bipolar disorder. During the follow-up period, 75 (5.80%) patients with bipolar disorder and 140 (2.71%) control patients were diagnosed with sexually transmitted diseases (*P* < .001). The most common subsequent sexually transmitted diseases in the patients with bipolar disorder were trichomoniasis in 28 patients (2.17%), genital warts in 14 patients (1.08%), and chlamydial infection in 13 patients (1.08%). Overall, significantly higher incidences of syphilis [11 (0.85%) versus 12 (0.23%), *P* < .001], genital warts [14 (1.08%) versus 13 (0.25%), *P* < .001], and trichomoniasis [28 (2.17%) versus 57 (1.10%), *P* = .006] were observed in the patients with bipolar disorder than in the control patients.

**Table 1:** Characteristics of bipolar disorder and sexually transmitted diseases (STIs) and control subjects

	Bipolar disorder	Control cohort	*P* values
No.	1,293	5,172	
Age (years)^a^	42.3 (30.9–56.3)	42.3 (30.9–56.3)	> .999
Distribution of age			
20–39	574 (44.39)	2,296 (44.39)	
40–64	512 (39.60)	2,048 (39.60)	
≥65	207 (16.00)	828 (16.00)	
Gender			> .999
Female	636 (49.19)	2,544 (49.19)	
Male	657 (50.81)	2,628 (50.81)	
Comorbidities			
Hypertension	380 (29.39)	1,115 (21.56)	< .001^*^
Diabetes mellitus	233 (18.02)	664 (12.84)	< .001^*^
Dyslipidemia	238 (18.41)	820 (15.85)	.029^*^
Coronary artery disease	234 (18.10)	671 (12.97)	< .001^*^
Congestive heart failure	59 (4.56)	143 (2.76)	.002^*^
Cerebrovascular disease	212 (16.40)	476 (9.20)	< .001^*^
Chronic pulmonary disease	205 (15.85)	543 (10.50)	< .001^*^
Income			< .001^*^
High income	108 (8.35)	583 (11.27)	
Medium income	197 (15.24)	1,001 (19.35)	
Low income	721 (55.76)	2,554 (49.38)	
No income	267 (20.65)	1,034 (19.99)	
Degree of urbanization			< .001^*^
Urban	687 (53.13)	3,113 (60.19)	
Suburban	396 (30.63)	1,692 (32.71)	
Rural	210 (16.24)	367 (7.10)	
Newly diagnosed STI, n (%)	75 (5.80)	140 (2.71)	< .001^*^
HIV infection	5 (0.39)	6 (0.12)	0.050
Syphilis	11 (0.85)	12 (0.23)	< .001^*^
Genital warts	14 (1.08)	13 (0.25)	< .001^*^
Gonorrhea	8 (0.62)	23 (0.44)	.376
Chlamydial infection	13 (1.01)	33 (0.64)	.193
Trichomoniasis	28 (2.17)	57 (1.10)	.006^*^
Follow-up, years^a^	10.68 (9.20–12.38)	11.02 (9.56–12.50)	< .001^*^

STI sexually transmitted diseases; HIV human immunodeficiency virus

^a^Median (interquartile range); ^*^Statistical significance

A subanalysis based on the duration of follow-up revealed that most of the STIs developed beyond the first year following a bipolar diagnosis and that the risk of syphilis, genital warts, and trichomoniasis remained significantly elevated when the patients diagnosed with STIs within 1 year of bipolar diagnosis were excluded. The results of the subanalysis are summarized in Table 2.

**Table 2:** Number of newly diagnosed sexually transmitted diseases between bipolar disorder and control subjects which was stratified by follow-up duration

Follow-up duration (years)	Bipolar disorder			Control cohort			Risk ratio (95% CI)
	No. of HIV infection	Per 1,000 person-years		No. of HIV infection	Per 1,000 person-years		
Overall	5	2.55		6	0.72		3.51 (0.85–13.81)
0–1	0	0		1	8.11		0.00 (0.00–69.24)
≥ 1	5	2.55		5	0.71		4.22 (0.97–18.33)
**Follow-up duration (years)**	**Bipolar disorder**			**Control cohort**			**Risk ratio (95% CI)**
	**No. of Syphilis**	**Per 1,000 person-years**		**No. of Syphilis**	**Per 1,000 person-years**		
Overall	11	4.68		12	1.10		3.87 (1.55–9.58)^*^
0–1	3	67.18		1	18.96		5.10 (0.41–267.70)
≥ 1	8	4.56		11	1.09		3.07 (1.07–8.39)^*^
**Follow-up duration (years)**	**Bipolar disorder**			**Control cohort**			**Risk ratio (95% CI)**
	**No. of Genital warts**		**Per 1,000 person-years**	**No. of Genital warts**	**Per 1,000 person-years**		
Overall	14		7.08	13	1.40		4.55 (1.98–10.50)^*^
0–1	1		6.97	0	0		N/A
≥ 1	13		7.08	13	1.40		4.23 (1.80–9.89)^*^
**Follow-up duration (years)**	**Bipolar disorder**			**Control cohort**			**Risk ratio (95% CI)**
	**No. of Gonorrhea**	**Per 1,000 person-years**		**No. of Gonorrhea**	**Per 1,000 person-years**		
Overall	8	1.89		23	1.31		1.47 (0.57–3.40)
0–1	1	37.10		2	34.92		0.89 (0.02–17.13)
≥ 1	7	1.83		21	1.29		1.41 (0.51–3.43)
**Follow-up duration (year)**	**Bipolar disorder**			**Control cohort**			**Risk ratio (95% CI)**
	**No. of Chlamydial infection**	**Per 1,000 person-years**		**No. of Chlamydial infection**		**Per 1,000 person-years**	
Overall	13	5.03		33		3.27	1.66 (0.80–3.24)
0–1	2	73.92		5		52.05	0.70 (0.07–4.27)
≥ 1	11	4.91		28		3.23	1.66 (0.75–3.44)
**Follow-up duration (year)**	**Bipolar disorder**			**Control cohort**			**Risk ratio (95% CI)**
	**No. of Trichomoniasis**	**Per 1,000 person-years**		**No. of Trichomoniasis**		**Per 1,000 person-years**	
Overall	28	9.17		57		5.09	2.09 (1.28–3.34)^*^
0–1	1	103.62		9		115.32	0.20 (0.00–1.45)
≥ 1	27	8.99		48		4.99	2.39 (1.44–3.91)^*^

HIV human immunodeficiency virus; CI confidence interval.

^*^Statistical significance.

In addition, a Cox proportional hazards regression analysis was conducted to calculate the HR of new STI diagnosis for the patients with bipolar disorder compared with the matched control subjects. The results revealed that patients with bipolar disorder exhibited a markedly higher risk for subsequent STIs (HR = 2.23, 95% confidence interval (CI) 1.68–2.96; Table 3). Furthermore, among patients with bipolar disorder, female gender was a risk factor for acquisition of STIs compared with male gender (HR = 2.36, 95% CI 1.77–3.16; Table 4). For individual STIs, the results indicated that the patients with bipolar disorder exhibited a markedly higher risks of subsequent syphilis (HR = 3.99, 95% CI 1.72–9.28), genital warts (HR = 5.17, 95% CI 2.37–11.29), and trichomoniasis (HR = 2.13, 95% CI 1.34–3.38; Table 5). Subgroups based on gender revealed that female patients with bipolar disorder exhibited higher risks of subsequent syphilis (HR = 7.76, 95% CI 1.86–32.43), genital warts (HR = 5.46, 95% CI 2.13–14.03), and trichomoniasis (HR = 2.30, 95% CI 1.44–3.67) than the matched female control subjects. However, male patients with bipolar disorder exhibited a higher risk than the matched male control subjects only for subsequent genital warts (HR = 4.34, 95% CI 1.11–17.01; Table 6A and 6B).

**Table 3:** Analyses of risk factors for sexually transmitted diseases in patients with and without bipolar disorder

Predictive variables	Univariate analysis		Multivariate analysis		
	HR (95% CI)	*P* value	HR (95% CI)		*P* value
Bipolar disorder	2.29 (1.73–3.04)	<.001		2.23 (1.68–2.96)	<.001^*^
Age (<40 = 1, ≥40 = 0)	1.41 (1.01–1.84)	.013		1.23 (0.93–1.65)	.153
Gender (Female = 1, Male = 0)	2.36 (1.76–3.16)	<.001		2.36 (1.76–3.16)	<.001^*^
Comorbidities					
Hypertension	0.63 (0.43–0.93)	.019		0.73 (0.47–1.12)	.144
Diabetes mellitus	1.07 (0.72–1.59)	.742			
Dyslipidemia	0.71 (0.46–1.07)	.100			
Coronary artery disease	0.78 (0.50–1.22)	.779			
Congestive heart failure	0.85 (0.32–2.28)	.743			
Cerebrovascular disease	0.66 (0.37–1.15)	.141			
Chronic pulmonary disease	1.10 (0.72–1.70)	.656			
Degree of urbanization					
Urban	Reference			Reference	
Suburban	0.81 (0.59–1.10)	.175		0.85 (0.62–1.12)	.293
Rural	1.61 (1.01–2.41)	.020		1.51 (1.01–2.28)	.047^*^
Income group					
No income	Reference				
Low income	1.10 (0.77–1.58)	.595			
Medium income	1.19 (0.78–1.81)	.422			
High income	0.73 (0.42–1.30)	.284			

HR indicates hazard ratio; CI indicates confidence interval

**Table 4:** Analyses of risk factors for sexually transmitted diseases in patients with bipolar disorder

Predictive variables	Univariate analysis			Multivariate analysis	
	HR (95% CI)	*P* value		HR (95% CI)	*P* value
Age (<40 = 1, ≥40 = 0)	1.13 (0.72–1.78)	.600			
Gender (Female = 1, Male = 0)	2.91 (1.73–4.89)	<.001	2.36 (1.77–3.16)		<.001^*^
Comorbidities					
Hypertension	0.55 (0.30–1.03)	.062	0.67 (0.45–1.01)		.057
Diabetes mellitus	0.54 (0.25–1.18)	.125			
Dyslipidemia	0.47 (0.22–1.03)	.060	0.80 (0.52–1.25)		.327
Coronary artery disease	0.75 (0.37–1.50)	.411			
Congestive heart failure	0.43 (0.06–3.10)	.403			
Cerebrovascular disease	0.66 (0.30–1.44)	.300			
Chronic pulmonary disease	1.31 (0.70–2.42)	.396			
Degree of urbanization					
Urban	Reference				
Suburban	0.65 (0.37–1.14)	.131			
Rural	1.01 (0.54–1.87)	.977			
Income group					
No income	Reference				
Low income	0.91 (0.50–1.66)	.767			
Medium income	1.01 (0.48–2.13)	.970			
High income	(0.48–2.66)	.786			

HR indicates hazard ratio; CI indicates confidence interval

**Table 5:** Hazard ratios of time until sexually transmitted diseases between bipolar disorder and control subjects during a ten-year follow-up period

	Crude HR (95% CI)	Adjusted HR (95% CI)^a^
HIV infection	3.53 (1.08–11.55)^*^	2.58 (0.73–9.11)
Syphilis	3.85 (1.70–8.73)^*^	3.99 (1.72–9.28)^*^
Genital warts	4.57 (2.15–9.73)^*^	5.17 (2.37–11.29)^*^
Gonorrhea	1.46 (0.65–3.25)	0.94 (0.41–2.16)
Chlamydial infection	1.66 (0.87–3.16)	1.57 (0.51–3.01)
Trichomoniasis	2.08 (1.32–3.27)^*^	2.13 (1.34–3.38)^*^

HIV human immunodeficiency virus; HR hazard ratio; CI confidence interval.

^*^Statistical significance; ^a^Adjusted for age, gender, hypertension, diabetes mellitus, dyslipidemia, coronary artery diseases, congestive heart failure, chronic pulmonary diseases, cerebrovascular diseases, income and urbanization.

**Table 6A:** Hazard ratios of time until sexually transmitted diseases between male patients with bipolar disorder and male control subjects during a ten-year follow-up period

	Crude HR (95% CI)	Adjusted HR (95% CI)^a^
HIV infection	2.90. (0.82–10.27)	2.25 (0.59–8.63)
Syphilis	2.39 (0.80–7.12)	2.76 (0.89–8.53)
Genital warts	3.50 (0.94–13.02)	4.34 (1.11–17.01)^*^
Gonorrhea	1.43 (0.46–4.43)	0.96 (0.30–3.10)
Chlamydial infection	0.94 (0.20–4.35)	0.73 (0.14–3.73)

HIV human immunodeficiency virus; HR hazard ratio; CI confidence interval.

^*^Statistical significance; ^a^Adjusted for age, gender, hypertension, diabetes mellitus, dyslipidemia, coronary artery diseases, congestive heart failure, chronic pulmonary diseases, cerebrovascular diseases, income and urbanization.

**Table 6B:** Hazard ratios of time until sexually transmitted diseases between female patients with bipolar disorder and female control subjects during a ten-year follow-up period

	Crude HR (95% CI)	Adjusted HR (95% CI)^a^
HIV infection	1291.59 (0.01–1.63E18)	83.20 (0.01–1.29E8)
Syphilis	8.21 (2.05–32.84)^*^	7.76 (1.86–32.43)^*^
Genital warts	5.16 (2.04–13.08)^*^	5.46 (2.13–14.03)^*^
Gonorrhea	1.49 (0.48–4.68)	1.02 (0.31–3.35)
Chlamydial infection	1.90 (0.93–3.88)	1.88 (0.91–3.88)
Trichomoniasis	2.30 (1.45–3.64) ^*^	2.30 (1.44–3.67)^*^

HIV human immunodeficiency virus; HR hazard ratio; CI confidence interval.

^*^Statistical significance; ^a^Adjusted for age, gender, hypertension, diabetes mellitus, dyslipidemia, coronary artery diseases, congestive heart failure, chronic pulmonary diseases, cerebrovascular diseases, income and urbanization.

## DISCUSSION

In our work, the prevalence of STIs, including HIV infection (0.39%), primary, secondary, and latent syphilis (0.85), genital warts (1.08%), gonorrhea (0.62%), chlamydial infection (1.01%), and trichomoniasis (2.17%), in patients with bipolar disorder were demonstrated. Compared with previous studies regarding the prevalence of STIs in patients with mental disorders, the prevalence of STIs in the current study was relatively low. For example, a study surveying the prevalence of STIs in psychiatric outpatients revealed prevalence of gonorrhea, chlamydial infection, and trichomoniasis of 1%, 3.3%, and 15.7%, respectively [10]. Furthermore, prevalence rates of syphilis in patients with mental illness varied from 1.1% to 7.6% [19]. A study reported that over one-third of the sample (38%) reported a lifetime history of one or more STIs in psychiatric outpatients [20]. There are two possible reasons for this discrepancy. First, underestimation of the prevalence of STIs in the current study should be considered. Because STIs may be asymptomatic, some patients with STIs may not have sought medical treatment and thus were not diagnosed. Moreover, patients with STIs may feel stigmatized because of their STIs and thus do not seek medical help. Second, most of previous studies results were based on cross-sectional study designs instead of a longitudinal design like our study.

In this study, we found that bipolar disorder was significantly associated with an increased sequential risk of STIs. There are several possible explanations for the increased risk of STIs in patients with bipolar disorder. First, impulsivity, hypersexuality, and impaired judgment are major symptoms of bipolar disorder, and these symptoms place patients with bipolar disorder at risk for STIs [21]. Second, a previous study demonstrated that patients bipolar disorder have high rates of risky sexual behaviors [22, 23]. For example, their compliance with contraceptive measures was poor [23]. Furthermore, they often engage in sex trading and are susceptible to engaging in coerced sex [22, 24]. Third, behavioral addictions, including sex addiction, are more frequent in patients with bipolar disorder than in healthy control subjects and are related to higher impulsivity levels and immaturity [25]. In a study investigating sexual behavior in a population of psychiatric patients with schizophrenia, schizoaffective disorder, and bipolar disorder, the results showed that most of the patients engaged in sexual behavior putting them at high risk for acquisition and transmission of STIs [23]. Finally, substance use disorders are common comorbidities in patients with bipolar disorder [21], and previous work has demonstrated that substance abuse and dependence increase the risk of STIs [26–28].

A previous study showed that female gender is a risk factor for STIs in the general population [29]. Our study demonstrates that female gender, compared with male gender, is also a risk factor for acquisition of STIs in patients with bipolar disorder. Higher rates of sexually risky behaviors in female patients with bipolar disorder than in male patients with bipolar disorder might be a possible explanation for this finding. In a study summarizing research pertinent to the clinical care of women with bipolar disorder, the results showed that women with bipolar disorder are more likely than men with bipolar disorder to report sex trading, resulting in a higher risk for STIs in female patients with bipolar disorder [22]. Furthermore, a lack of female contraceptive devices also adds to the vulnerability of these patients [24].

In the present study, we conducted a subgroup analysis stratified according to the duration between the diagnosis of bipolar disorder and new-onset STIs (Table 2). The results indicate that incident syphilis, genital warts, and trichomoniasis were increased beyond the first year following a bipolar diagnosis. Patients with bipolar disorder are likely to exhibit a higher frequency of outpatient and inpatient visits than the general population, leading to an earlier diagnosis of STIs, which cause surveillance bias. However, in our work, the risk ratio for the newly diagnosed syphilis, genital warts, and trichomoniasis did not increase within 1 year of bipolar diagnosis in the bipolar cohort. Moreover, when patients diagnosed with STIs within 1 year of bipolar diagnosis were excluded, the risk ratio for newly diagnosed syphilis, genital warts, and trichomoniasis remained high for the bipolar cohort, and the ratios were all statistically significant. This result suggests that the increased risks of syphilis, genital warts, and trichomoniasis in patients with bipolar disorder were not caused by surveillance bias.

This study has several strengths. First, our study design included an unbiased patient selection process. Because participation in the NHI is mandatory and all residents of Taiwan can access health care with low copayments, referral bias is low, and follow-up compliance is high. Second, our study is a population-based study including large samples from all hospitals across the country. In studies with small samples or in single-hospital case studies, which are common in the relevant literature, it is difficult to develop such an index with high acceptability across the health care industry. Third, data used in this study were derived from the NHI system in Taiwan. As an observational database, these data reflect current real-world diagnostic patterns.

However, the present study has several limitations inherent in the use of claims databases that should be considered. First, the NHIRD does not provide detailed information on patients, such as number of sex partners, engaging in sex with promiscuous partners, and condom use. These are all significant risk factors for STIs. Thus, we were unable to control for these potentially confounding factors. Second, compared with previous studies regarding the prevalence of STIs in patients with mental disorders, the prevalence of STIs in the current study was relatively low. Underestimation of the prevalence of STIs in the current study should be considered. Third, information on the diagnostic accuracy and severity of bipolar disorder in the current study were unavailable. Whether the severity of bipolar disorder is related to STI development should be further evaluated.

In conclusion, this nationwide cohort study indicated an increased risk of syphilis, genital warts, and trichomoniasis among patients with bipolar disorder. Female gender is a risk factor for acquisition of STIs in patients with bipolar disorder. Taking a sexual history from persons with bipolar disorder is important, and patients engaging in high-risk sexual behavior should be routinely screened for STIs to enable detection, treatment, and preventive education. Furthermore, prospective studies, particularly those with additional patient-level data, are warranted to confirm our findings.

## PATIENTS AND METHODS

### Data sources

Established in 1995, the National Health Insurance (NHI) program is a mandatory health insurance program that offers comprehensive medical care coverage, including outpatient, inpatient, emergency, and traditional Chinese medicine, to all residents of Taiwan, with a coverage rate of up to 98% [30]. The NHI Research Database (NHIRD) contains comprehensive information regarding clinical visits, including prescription details and diagnosis codes based on the A code and International Classification of Diseases, Ninth Revision, Clinical Modification (ICD-9-CM). The NHIRD is managed and publicly released by the National Health Research Institutes (NHRI) for research purposes, and confidentiality is maintained according to the directives of the Bureau of NHI. The data source for our study was the Longitudinal Health Insurance Database 2000 (LHID 2000), which is a dataset included in the NHIRD. Data for the LHID was collected by systematically and randomly sampled from the NHIRD; this database includes the data of 1 million individuals. The NHRI reported no significant differences in sex distribution, age distribution, or average insured payroll-related amount between the patients in the LHID and those in the original NHIRD [31].

### Ethics statement

The Institutional Review Board of Taichung Veterans General Hospital approved this study. Written consent from the study patients was not obtained because the NHI dataset consists of de-identified secondary data for research purposes and the Institutional Review Board of Taipei Veterans General Hospital issued a formal written waiver for the need for consent.

### Study population

Using data extracted from the LHID 2000, we conducted a retrospective cohort study of patients who were newly diagnosed with bipolar disorder between January 1, 2000, and December 31, 2004. The patients with bipolar disorder were defined as meeting criteria for ICD-9-CM codes 296.0, 296.1, 296.4, 296.5, 296.6, 296.7, 296.8, 296.80, or 296.89. We excluded patients who were diagnosed with bipolar disorder between January 1, 1996, and December 31, 1999. We also excluded patients who were diagnosed with STIs (HIV infection; primary, secondary, and latent syphilis; genital warts; gonorrhea; chlamydial infection; and trichomoniasis) before they were diagnosed with bipolar disorder. For each patient with bipolar disorder included in the final cohort, four age- and gender-matched control patients without STIs were randomly selected from the LHID 2000. All patients with bipolar disorder and all control subjects were observed until diagnosed with HIV infection (ICD-9-CM codes 042 or V08, A049); primary, secondary, and latent syphilis (ICD-9-CM codes 091–097, A060); genital warts (ICD-9-CM code 078.11); gonorrhea (ICD-9-CM codes 098, A061); chlamydial infection (ICD-9-CM codes 078.8, 078.88); or trichomoniasis (ICD-9-CM codes 131, A079). If they did not meet any of these criteria, they were observed until death, withdrawal from the NHI system, or December 31, 2013. The primary clinical outcomes assessed were newly diagnosed HIV infection; primary, secondary, and latent syphilis; genital warts; gonorrhea; chlamydial infection; and trichomoniasis. Common comorbidities, including hypertension, diabetes mellitus, dyslipidemia, coronary artery disease, congestive heart failure, chronic pulmonary disease, and cerebrovascular disease, were also compared between patients with bipolar disorder and control subjects. Insurance premiums, calculated according to the beneficiary’s total income, were used to estimate monthly income. Monthly income was grouped into no income, low income (monthly income < 20,000 New Taiwan Dollar [NTD]), median income (20,000 NTD ≤ monthly income < 40,000 NTD), and high income (monthly income ≥ 40,000 NTD). Urbanization was divided into three groups: urban, suburban, and rural. Urbanization and monthly income levels were used to represent socioeconomic status.

### Statistical analysis

The primary outcomes in this study were occurrence of newly diagnosed HIV infection; primary, secondary, and latent syphilis; genital warts; gonorrhea; chlamydial infection; or trichomoniasis. We first compared the distribution of demographic characteristics between the patients with bipolar disorder and control subjects by using independent *t*-tests and chi-squared tests. To investigate potential surveillance bias, subgroups were stratified according to the duration of enrollment. In addition, a Cox proportional hazards regression model was used to identify the risk factors associated with STIs in the whole sample and among patients with bipolar disorder. Control variables, such as age; sex; common comorbidities, including hypertension, diabetes mellitus, dyslipidemia, coronary artery disease, congestive heart failure, chronic pulmonary disease, and cerebrovascular disease; urbanization; and monthly income were included as covariates in the univariate model. Factors that demonstrated a moderately significant statistical relationship in the univariate analysis (*P* < .1) were entered by forward selection in a multivariate Cox proportional-hazards regression model. Furthermore, the Cox proportional hazards regression model was also constructed to calculate the hazard ratio (HR) of six different kinds of STIs, including HIV infection; primary, secondary, and latent syphilis; genital warts; gonorrhea; chlamydial infection; and trichomoniasis. Subgroups were also stratified according to gender to survey the risk of STIs of male and female subjects in the bipolar and control cohorts.

The SAS statistical software for Windows, Version 9.3 (SAS Institute, Cary, NC, USA), was used for data extraction, computation, linkage, processing, and sampling. All other statistical analyses were performed using the SPSS statistical software for Windows, Version 20 (IBM, Armonk, NY, USA). The results of comparisons with a *P* value less than .05 were considered to indicate a statistically significant relationship.

## Abbreviations

STIs: sexually transmitted infections
HR: hazard ratio
CI: confidence interval
HIV: human immunodeficiency virus
NHI: national health insurance
NHIRD: national health insurance research database
ICD-9-CM: International Classification of Diseases
Ninth Revision: Clinical Modification
NHRI: the National Health Research Institutes
LHID 2000: the Longitudinal Health Insurance Database 2000
